# Posterior Corneal Surface Stability after Femtosecond Laser-Assisted Keratomileusis

**DOI:** 10.1155/2015/184850

**Published:** 2015-09-21

**Authors:** Carlo Cagini, Marco Messina, Marco Lupidi, Francesco Piccinelli, Tito Fiore, Daniela Fruttini, Leopoldo Spadea

**Affiliations:** ^1^Department of Surgery and Biomedical Science, University of Perugia and S. Maria della Misericordia Hospital, Sant'Andrea delle Fratte, 06156 Perugia, Italy; ^2^Centre Ophtalmologique de l'Odéon, 113 Boulevard Saint-Germain, 75006 Paris, France; ^3^Department of Economics, Finance and Statistics, University of Perugia, 06156 Perugia, Italy; ^4^Department of Biotechnology and Medical-Surgical Sciences, “Sapienza” University of Rome, 04100 Latina, Italy

## Abstract

The purpose of this study was to evaluate posterior corneal surface variation after femtosecond laser-assisted keratomileusis in patients with myopia and myopic astigmatism. Patients were evaluated by corneal tomography preoperatively and at 1, 6, and 12 months. We analyzed changes in the posterior corneal curvature, posterior corneal elevation, and anterior chamber depth. Moreover, we explored correlation between corneal ablation depth, residual corneal thickness, percentage of ablated corneal tissue, and preoperative corneal thickness. During follow-up, the posterior corneal surface did not have a significant forward corneal shift: no significant linear relationships emerged between the anterior displacement of the posterior corneal surface and corneal ablation depth, residual corneal thickness, or percentage of ablated corneal tissue.

## 1. Introduction

Laser-assisted in situ keratomileusis (LASIK) is the most common surgical technique for correcting refractive defects and although its efficacy and safety have already been proven, it is not free of potential complications. One of the most feared is postoperative ectasia which induces astigmatism and myopia and reduces the quality of vision due to the induced high-order aberrations and in severe cases it reduces visual acuity. In order to detect ectatic change early, many authors have studied posterior corneal surface stability after laser refractive surgery [1, 2]. Although postoperative ectasia was described in several papers [1, 3], its cause remains unclear. Onset seems to be favored by conditions such as a low residual corneal thickness, an abnormal preoperative corneal topography, a high refractive defect, or the patient's young age [3–5].

Today femtosecond laser (FSL) is widely used in LASIK surgery to create the corneal flap. Compared with the microkeratome (MK), it has increased the safety of the operation and improved the predictability and homogeneity of the corneal flap thickness [6]. The latter has been shown to limit loss of corneal tensile strength especially for thin-flap LASIK (90–120 *μ*m) [7].

To our knowledge, no studies have as yet focused on posterior corneal surface changes after femtosecond laser-assisted keratomileusis (FSL-LASIK) using a corneal tomography unit. The purpose of the present study is to evaluate the posterior corneal surface in eyes which underwent FSL-LASIK for myopia and myopic astigmatism over a twelve-month follow-up period in order to highlight any eventual postoperative forward shift.

## 2. Materials and Methods

After the local ethics committee had approved the study protocol, patients were enrolled in a prospective noncomparative case series between January 2012 and June 2012. Inclusion criteria were age of 21 years and older with stable myopia and a myopic astigmatism for at least two years. Exclusion criteria were systemic or ocular pathologies interfering with the healing process of the cornea, keratoconus, corneal dystrophy, glaucoma, previous eye trauma, or surgery.

The study was conducted on patients undergoing FSL-LASIK at the Department of Surgery and Biomedical Science of University of Perugia. All patients underwent a complete preoperative assessment, including corneal tomography using a Scheimpflug camera (Sirius, C.S.O. Costruzione Strumenti Oftalmici, Florence, Italy). Four tomographic images were obtained for each eye, and the best image was chosen. This instrument acquires a series of 25 Scheimpflug images and a single Placido image. Anterior corneal surface data are derived from the integration of both types of images, while data on the posterior corneal surface and the lens are derived only from Scheimpflug images. Image integration and processing were done using Phoenix Software (version 1.0.5.72).

*Surgery*. A corneal flap of 9.0 mm in diameter was made at a depth of 110 microns with the femtosecond laser (VisuMax, Carl Zeiss Meditec AG, Jena, Germany). Once the corneal flap was lifted, photoablative excimer laser treatment was applied (MEL 80, Carl Zeiss Meditec AG, Jena, Germany).

Check-ups were scheduled for 1, 6, and 12 months after surgery. At each check-up changes in the posterior corneal curvature (PCC), posterior corneal elevation (PCE), with respect to the best-fit sphere, and the anterior chamber depth as evaluated from the endothelium (ACD) were studied by comparing the results of the preoperative evaluation with postoperative data. The postoperative forward shift was expressed as the difference between preoperative and postoperative PCC values in the central 4 mm areas of the elevation maps, assigning a positive value to anterior shift. All examinations were performed by the same experienced operator (F.P.). Differences in posterior corneal curvature, ablation, residual thickness, and the ablation percentage of the total preoperative corneal thickness were analyzed by Spearman's coefficient of rank correlation (*ρ*). The analysis of variance (ANOVA) for repeated measures and Student's *t*-test for paired data with the Bonferroni correction were used for data analysis. Statistical significance was set at *p* ≤ 0.05. The software was SAS version 9.2.

## 3. Results

Forty-two eyes of 42 patients underwent bilateral and simultaneous FSL-LASIK surgery, and one eye of each subject was randomly chosen (coin toss) and included in this study. The mean age of patients was 36.05 ± 8.3 years (range: 24–53 years). The mean preoperative spherical equivalent was −5.05 ± 2.07 D (range: −1.25 D/−7.50 D) and the programmed ablation depth was 117.2 ± 32.2 *μ*m (36–160).

Postoperative corneal haze degree was under 1 in all eyes.  Table 1 reports mean values of the posterior corneal curvature, posterior corneal elevation, and anterior chamber depth. Data analysis detected no significant changes in the posterior corneal curvature at 1, 6, and 12 months after surgery compared with preoperative values (*F* = 1.47; *p* = 0.226). There was no significant change in posterior corneal elevation during the 12-month follow-up period (*F* = 1.89; *p* = 0.135). Anterior chamber depth was stable 1 month and 6 months after FSL-LASIK surgery but a significant reduction was observed 12 months after surgery (0.03 ± 0.07 mm) (*F* = 4.25; *p* = 0.007) (Bonferroni test: *t* = 2.94; *p* = 0.005).

**Figure 1:**
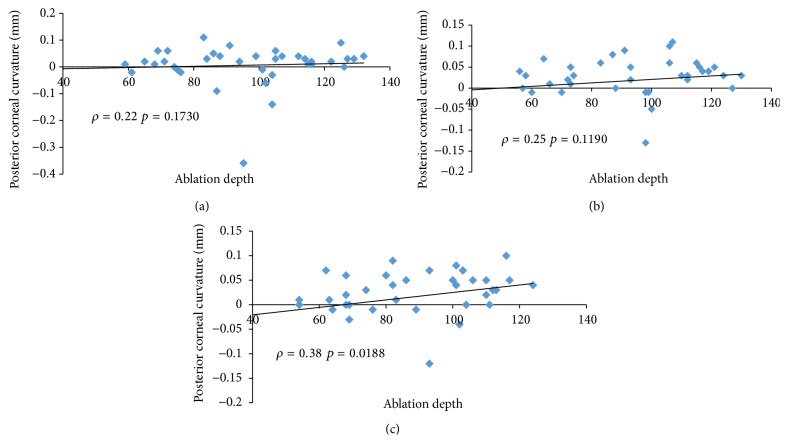
Spearman's coefficient of rank correlation (*ρ*) between corneal ablation depth and changes in posterior corneal curvature (mm) at 1 month (a), 6 months (b), and one year (c).

**Figure 2:**
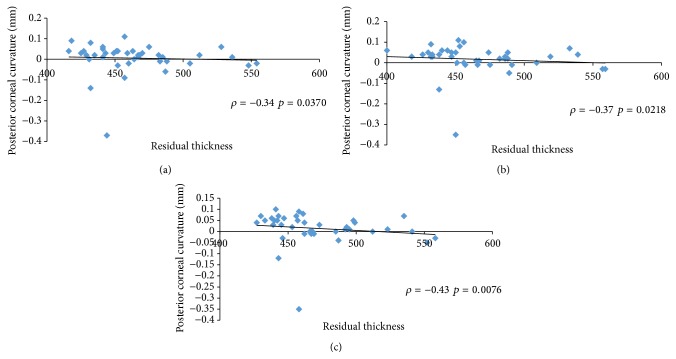
Spearman's coefficient of rank correlation (*ρ*) between residual corneal thickness and changes in posterior corneal curvature (mm) at 1 month (a), 6 months, (b) and one year (c).

**Figure 3:**
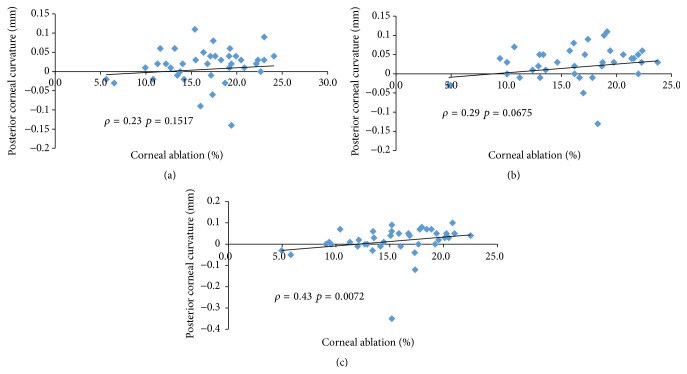
Spearman's coefficient of rank correlation (*ρ*) between percentage of corneal ablation and changes in posterior corneal curvature (mm) at 1 month (a), 6 months (b), and one year (c).

Our data did not show any linear correlation between corneal ablation depth and posterior corneal curvature throughout the follow-up period (Figures 1(a), 1(b), and 1(c)). No relationships emerged between posterior corneal curvature, residual corneal thickness (Figures 2(a), 2(b), and 2(c)), and the percentage of ablated corneal tissue (Figures 3(a), 3(b), and 3(c)).

## 4. Discussion

Our study on patients undergoing FSL-LASIK for myopia and myopic astigmatism shows the posterior corneal surface was stable over time. Since refractive surgery reduces corneal thickness, weakening of this layer might possibly have a modification of the posterior corneal surface. In fact, one potential complication of this type of surgery is posterior corneal ectasia which ranges in frequency from 0.04 to 0.9% depending on several factors such as the accuracy of the preoperative evaluation, the surgical technique, and the surgeon's skill [3, 5, 8, 9].

Using Orbscan (Orbscan, Bausch & Lomb, Tampa, FL, USA) a forward shift of the posterior corneal surface was observed after photokeratectomy (PRK) and LASIK [1, 4, 10]. Orbscan sensitivity in studying the posterior corneal surface after refractive surgery was, however, questioned because a change in optical features does not allow the instrument to provide accurate corneal mapping [11, 12]. Orbscan uses mathematical calculations to recreate the posterior corneal surface and this strategy, in patients undergoing refractive surgery, can cause false positive readings of the posterior corneal elevation.

Corneal morphology is assessed better by rotating Scheimpflug cameras that analyze the posterior corneal elevation without mathematical calculations [13]. Evaluations by Pentacam (Oculus GmbH, Wetzlar, Germany), which is the first instrument to be based on this technology, appear more reliable as they seem less influenced by the effects of refractive surgery. The different sensitivities of the Scheimpflug camera and Orbscan in studying the corneal morphology were demonstrated in 2007 by Nishimura et al. [12] and later confirmed by others [14]. Studies conducted with Pentacam showed that the risk of presenting a forward displacement of the posterior corneal surface after refractive surgery was lower than that which had been suggested using Orbscan. In using Pentacam to monitor myopic eyes undergoing PRK or MK-LASIK, Ciolino and Belin [11] did not observe any case of forward shift at 2 months after surgery. This observation was confirmed by Nishimura et al. [12] and by Sun et al. [2] in patients undergoing PRK or Epi-LASIK at 3 months after surgery. Although reduced, the forward shift complication did not completely disappear. In 2010, using Pentacam, Zhang and Wang [15] observed a forward shift of posterior corneal elevation in patients 1 month and 6 months after Epi-LASIK surgery. Nowadays, cases of iatrogenic corneal ectasia after LASIK are still reported even in absence of detectable preoperative risk factors in eyes studied with Orbscan or with the Scheimpflug camera [16, 17].

In our opinion, some limitations emerge from these reports. Most studies on posterior corneal surface stability after LASIK have short follow-ups [2, 11, 12], even though it is well known that a change in the corneal morphology may be observed even a long time after refractive surgery has been performed [1]. Moreover, many studies were conducted on patients who underwent LASIK using a microkeratome which does not ensure a high repeatability and regularity of the corneal cut to the detriment of flap thickness homogeneity. On the contrary, the femtosecond laser allows the cut to be made at a lower depth, with greater predictability of and homogeneity in thickness [18]. This probably reduces the loss of corneal tensile strength [6, 10] and should minimize the risk of postoperative ectasia. In a retrospective study with a long follow-up period, Moshirfar et al. [19] used Orbscan to study eyes undergoing FSL-LASIK and observed 0.05% incidence of iatrogenic ectasia and 0.25% total incidence of postoperative ectasia.

In our study, we used a recently introduced rotating Scheimpflug camera that was proven to be accurate and reliable in studies on healthy eyes [20] and on eyes affected by pathological ectasia [21] with high repeatability and reproducibility. Moreover, Sirius system demonstrated high repeatability and reproducibility for anterior chamber depth measurements [22, 23].

In our patients who underwent FSL-LASK, we did not observe any change in the posterior corneal surface even one year after surgery. To our knowledge, there are no other similar studies in the literature. Our findings concur with other authors who reported that a Scheimpflug camera after MK-LASIK demonstrated posterior corneal surface stability after a long follow-up period [2, 24]. Furthermore, in agreement with other authors [2, 11, 12], the difference in posterior corneal curvature in our study did not correlate with residual corneal thickness, corneal ablation depth, or the percentage of ablated corneal tissue.

LASIK surgery induces a change in the corneal morphology with an increase in peripheral corneal curvature and posterior central curvature without a forward shift [25]. Observed soon after surgery, these effects tend to decrease over time but can be confused with a bulge of the posterior corneal surface [26]. In our work, we did not observe any changes in the posterior corneal surface probably because our postoperative examinations began one month after surgery. Moreover, use of the femtosecond laser to create the corneal flap may have lessened these effects on corneal morphology.

Unexpectedly, we observed a reduction in the anterior chamber depth 1 year after surgery, without any clinical consequences, as was reported by Nishimura et al. [12] in 2007 and by Sun et al. [2] in 2009. This paradoxical observation may be one result of the magnification effect that the cornea has on the lens front surface after LASIK [27], or it may be caused by the change in lens morphology due to the increased accommodative effort that patients must make once the myopic refractive error is corrected [2]. In fact, its early appearance is a consequence of peripheral stromal thickening which recovers the normal morphology within three months, but we cannot account for its onset one year after surgery.

## 5. Conclusion

Using a rotating Scheimpflug camera, our study demonstrated posterior corneal surface stability 12 months after surgery in patients who underwent FSL-LASIK for myopia and myopic astigmatism. Although these data should be confirmed by further studies, with more patients, our work seems to confirm that the femtosecond laser plays a role in making this surgery safer and more predictable, probably by reducing the onset of forward shift of the posterior corneal surface.

## Figures and Tables

**Table 1:** Posterior corneal curvature (PCC), posterior corneal elevation (PCE), and anterior chamber depth (ACD) before FS-LASIK and 1 month, 6 months, and one year afterwards.

	Preoperative	Postoperative		
		1 month	6 months	1 year
PCC	6.54 ± 0.23 (mm)	6.53 ± 0.24 (mm)	6.52 ± 0.24 (mm)	6.52 ± 0.24 (mm)
PCE	10.77 ± 3.17 (*μ*m)	11.65 ± 3.44 (*μ*m)	11.87 ± 3.51 (*μ*m)	11.57 ± 3.04 (*μ*m)
ACD	3.37 ± 0.26^*∗*^ (mm)	3.35 ± 0.28 (mm)	3.36 ± 0.28 (mm)	3.34 ± 0.28^*∗*^ (mm)

^*∗*^ *p* < 0.05.
